# Tryptophan Catabolism and Inflammation: A Novel Therapeutic Target For Aortic Diseases

**DOI:** 10.3389/fimmu.2021.731701

**Published:** 2021-09-23

**Authors:** Tharmarajan Ramprasath, Young-Min Han, Donghong Zhang, Chang-Jiang Yu, Ming-Hui Zou

**Affiliations:** Center for Molecular and Translational Medicine, Georgia State University, Atlanta, GA, United States

**Keywords:** aortic aneurysm, atherosclerosis, kynurenine pathway, tryptophan metabolism, vascular cells

## Abstract

Aortic diseases are the primary public health concern. As asymptomatic diseases, abdominal aortic aneurysm (AAA) and atherosclerosis are associated with high morbidity and mortality. The inflammatory process constitutes an essential part of a pathogenic cascade of aortic diseases, including atherosclerosis and aortic aneurysms. Inflammation on various vascular beds, including endothelium, smooth muscle cell proliferation and migration, and inflammatory cell infiltration (monocytes, macrophages, neutrophils, etc.), play critical roles in the initiation and progression of aortic diseases. The tryptophan (Trp) metabolism or kynurenine pathway (KP) is the primary way of degrading Trp in most mammalian cells, disturbed by cytokines under various stress. KP generates several bioactive catabolites, such as kynurenine (Kyn), kynurenic acid (KA), 3-hydroxykynurenine (3-HK), etc. Depends on the cell types, these metabolites can elicit both hyper- and anti-inflammatory effects. Accumulating evidence obtained from various animal disease models indicates that KP contributes to the inflammatory process during the development of vascular disease, notably atherosclerosis and aneurysm development. This review outlines current insights into how perturbed Trp metabolism instigates aortic inflammation and aortic disease phenotypes. We also briefly highlight how targeting Trp metabolic pathways should be considered for treating aortic diseases.

## Introduction

Aortic diseases are the primary public health concern caused by age, genetics, diabetes, obesity, sedentary lifestyle, infection and injury. A slow and gradual thickening of the arteries, otherwise called atherosclerosis, is the common cause of cardiovascular diseases. Further, human arteries become less flexible with increased ages, leading to aortic stiffness or a partially dilated artery called aneurysm. Being asymptomatic, abdominal aortic aneurysm (AAA) is a common and potentially life-threatening condition as it may lead to rupture. However, elective aortic surgery is also associated with risks; elective repair of the aneurysm is the only way to prevent rupture. Thus, this condition requires improved pharmacologic interventions, which lacks in this modern medical system.

## Inflammation and Aortic Diseases (Atherosclerosis and AAA)

Atherosclerosis and AAAs are multifactorial and polygenic diseases with known environmental and genetic risk factors contributing to disease development (1, 2). Atherosclerosis is a chronic progressive inflammatory disorder that presents with coronary artery disease (CAD) (3). CAD accounts for approximately 610,000 deaths annually (estimated 1 in 4 deaths) and is the leading cause of mortality in the United States (3). AAAs are majorly caused by aging, hypertension, nicotine usage and atherosclerosis (4). Traditionally plenty of evidence showed atherosclerosis as a common etiology for thoracic aortic aneurysms (TAAs) and AAAs. AAA is a focal progressive dilatation of the aorta with a diameter of at least 50% greater than the average proximal diameter due to irreversible structural aortic wall integrity loss. It is one of the significant causes of worldwide morbidity and mortality that affects>1 million people in the United States alone (5). According to CDC, AAAs were the cause of 9,923 deaths in 2018 in the United States (6), and the mortality rate associated with AAA rupture is 88%. Given the high mortality and morbidity related to ruptured AAAs, this disease has traditionally posed a heavy burden on healthcare systems (7), and there have been only modest improvements in mortality over the last three decades. The pathogenesis of AAA includes endothelial cell (EC) dysfunction and vascular smooth muscle cells (VSMC) apoptosis/senescence. Endothelial and VSMCs dysfunction both can contribute to atherogenesis, which is widely accepted (8, 9). A variety of anti-inflammatory, antioxidant, beta-blockers and hemodynamic modulator drugs and matrix metalloproteinase (MMP) inhibitors are being studied to slow aneurysm growth (9). However, there are no pharmacological treatments available to either prevent or reverse the development of AAA.

Our increasing knowledge suggests that inflammatory processes are involved in the pathogenesis of aortic diseases (10). An imbalance between the production and release of proinflammatory factors has been reported in AAA’s pathology (11) and atherosclerosis progression (12). The native and adaptive immune responses initiate and propagate the inflammatory response to AAA pathology (13). During the development of AAA, infiltration of many exogenous immune cells, including lymphocytes, macrophages, mast cells, neutrophils, and natural killer cells infiltrate gradually into the tissue from adventitia to the intima, elicit a continuous inflammatory response (10). The massive inflammatory cells infiltration was interpreted in human aortic aneurysm surgical samples. These infiltrations are usually absent among the healthy aortic specimens. In AAA tissues, the B lymphocytes, T lymphocytes and macrophages were majorly characterized cell populations. Whereas the mast cells and natural killer cells were characterized as minor cell populations in these AAA tissues (14). These infiltrated Th1 mononuclear cells secrete the cytokines such as IL-2, IFNγ, and TNFα, to stimulate proinflammatory osteopontin secretion from macrophages that can propagate the inflammatory response during the AAA development (15). Besides the immune cell infiltration, factors released from dysfunctional perivascular aortic tissue (PVAT), including several cytokines and adipokines, could also contribute to arterial remodeling *via* immune activation.

## Tryptophan/Kynurenine Metabolism

L-Tryptophan (Trp) is an essential amino acid that should be obtained from dietary intakes such as vegetal (potatoes, chickpeas, soybeans, cocoa beans, and nuts) and animal origin (dairy products, eggs, meat, and seafood) (16, 17). The tryptophan is so crucial for protein synthesis and thus it is required for normal cellular homeostasis. It also serves as an *in vivo* precursor for several bioactive compounds, including nicotinamide (vitamin B6), serotonin, melatonin, tryptamine, and kynurenines (18). Hepatic tryptophan-2,3-dioxygenase (TDO) is known to play a critical role in keeping the physiological concentrations of Trp and kynurenine (Kyn) at a controlled level *via* kynurenine pathway (KP). In humans’ serum concentrations of Trp are in the range of 70 ± 10 µmol/L for males and 65 ± 10 µmol/L for females and Kyn concentrations are around 1.8 ± 0.4 µM and do not differ between genders (19). Considering the KP metabolisms and their significant association to many biological activities, the perturbations in the KP have been linked to several diseases.

**Figure 1:**
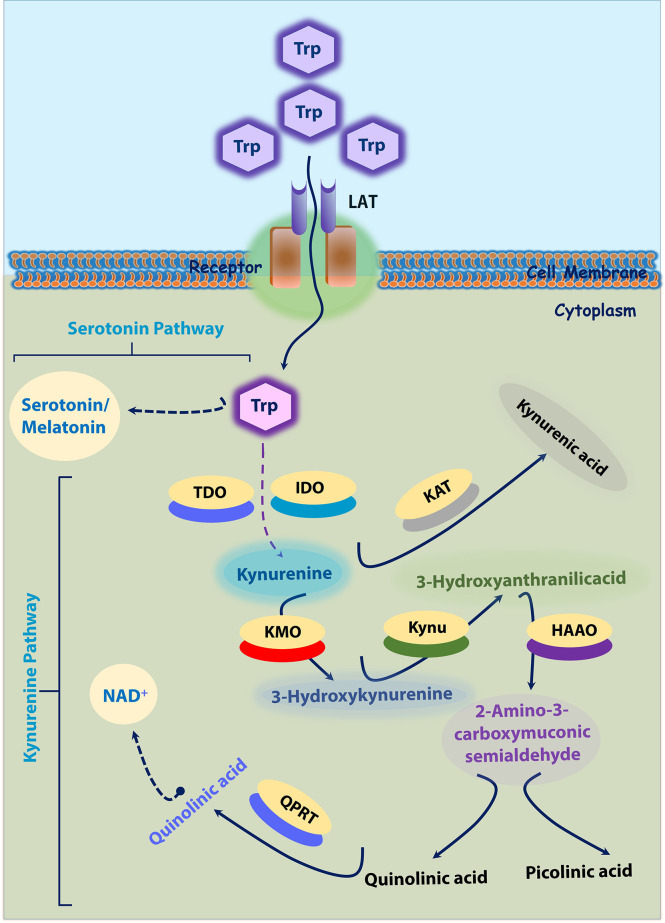
Tryptophan-Kynurenine and Serotonin Pathways. ~95% Trp is transported into cytoplasm by LAT. In cytoplasm, tryptophan is initially converted into kynurenine; Kynurenine into 3-Hydroxykynurenine by KMO; 3-Hydroxykynurenine into 3-Hydroxyanthranilicacid and further quinolinic acid or picolinic acid. In another axis, kynurenine is converted into kynurenic acid by KAT. In another hand, tryptophan (~5%) is converted into serotonin by serotonin pathway. 3-HK, 3-hydroxykynurenine; HAAO -3, hydroxyanthranilic acid dioxygenase; IDO, Indoleamine 2, 3-dioxygenase; KAT, kynurenine amino transferase; KAT, kynurenine amino transferase; KMO, kynurenine monooxygenase; Kynu, kynureninase; LAT, L-amino acid transporters; PA, picolinic acid; QA, quinolinic acid; QPRT, Quinolinate phosphoribosyl transferase; TDO, tryptophan-2,3-dioxygenase; Trp-tryptophan.

Two significant pathways that process Trp into other metabolites are serotonin and kynurenine pathways. Most dietary Trp (>95%) is fed into the KP, giving rise to several downstream metabolites (19, 20). The absolute and relative concentrations of kynurenines vary among different cell types due to different enzymatic repertoires (19). These Trp catabolites are activated in times of stress and inflammation (21). Three important rate-limiting enzymes indoleamine 2, 3-dioxygenase 1 and 2 (IDO1 and IDO2), and TDO utilize Trp as a substrate and generate N-formylkynurenine during the initial steps on Trp catabolism. This N-formylkynurenine is rapidly metabolized by formamidase into l-kynurenine (Kyn). Kyn is further catabolized into several potent metabolites such as 3-hydroxykynurenine (3-HK), 3-hydroxyanthranilic acid (3-HAA), kynurenic acid (KA), and xanthurenic acid (XA), quinolinic acid (QA), and produce the essential pyridine nucleotide end product, nicotinamide adenine dinucleotide^+^ (NAD^+^) (22). In brief, IDO produced Kyn further catabolized by kynureninase (Kynu) produces anthranilic acid (AA). Kynurenine-3-monooxygenase (KMO) also converts Kyn into 3-HK, which is further utilized by kynurenine aminotransferase (KAT) to produce XA or by the Kynu to form 3-HAA. Further, 3-HAA is converted into quinolinic acid (QA) or picolinic acid (PA) by a series of enzymatic conversions. In addition, KAT metabolizes Kyn into KA as well (**Figure 1**).

In hepatocytes TDO expression is stimulated by glucocorticoids. In contrast, the IDO1 present outside the liver is stimulated by proinflammatory cytokines (21). In the majority of cell types, IDO expression is induced by proinflammatory modulators, such as lipopolysaccharide (LPS), tumor necrosis factor-α (TNFα), interleukin 1 (IL-1), and IL-2 (23, 24). IDO isoforms, (IDO1 and IDO2) closely linked on chromosome 8 in humans, probably originating from an ancient gene duplication (23, 24). IDO1 is a heme-containing enzyme that catabolizes compounds containing indole rings, such as the essential amino acid Trp. The IDO1 isoform is expressed in various tissues, including dendritic cells, endothelial cells, macrophages, fibroblasts, and mesenchymal stromal cells, all are present in the arterial wall. This major isoform contributes to Trp degradation (25, 26). Transport of the amino acid l-tryptophan across the plasma membrane is known to occur through brush border L-amino acid transporters (LATs) (27). A well-known inducer interferon-gamma (IFN-γ) released from activated CD4+ T cells, robustly induces IDO1 expression contributing to Trp catabolism. IFN-γ coordinately induces LATs to maximize tryptophan depletion in IDO1-expressing cells and that the process involves a positive feedback mechanism *via* kynurenine-aryl hydrocarbon receptor (AhR) signaling (27). The IDO2 isoform is primarily expressed in the kidney, brain, colon, liver, and reproductive tract (25). Although the role of IDO1 is widely studied, the function of IDO2 is largely unexplored. Despite IDO1 and IDO2 exhibit critical functional differences, IDO2 was characterized as having a weaker catalytic activity than IDO1 *in vitro* (24). All these data strongly suggest the importance of the tissue-specific expression and localization of kynurenines producing proteins, which might regulate many signaling pathways and the body’s physiological status.

## Tryptophan Metabolism, Inflammation and Aortic Diseases

### Tryptophan Metabolism and Aortic Diseases

The altered amino acid metabolism and their metabolites were observed in the plasma of patients with AAA. Untargeted metabolic profiling of plasma showed statistically increased concentrations of amino acid metabolites in the plasma of people with large aneurysms when compared to the control population. Thus, beyond contributing to protein synthesis, amino acid metabolism plays a critical role in supporting various cell functions (28–30), which is positively as well as negatively correlated to vascular disease development. Among many amino acids, l-Arginine (Arg), l-homoarginine (hArg), and l-tryptophan (Trp) are important amino acids, and their metabolites have a putative role in determining cardiovascular diseases (31). For example, L-arginine, an essential amino acid, improves endothelial function and cardiovascular health (32). It is also known to alter inflammatory functions, and arginine supplementation has wound healing potential by reducing inflammation (33, 34).

Lines of evidence suggest that IDO1 and the Kyn pathway significantly contribute to cardiovascular diseases and thrombus formation. The incidence, development, and progression of vascular diseases are associated with body metabolism in general. Very importantly, accumulating evidence shows that Trp has a significant contribution to determine the AAA development. Trp metabolism, otherwise known as KP, is dysregulated during vascular inflammation and many cardiovascular diseases. A study conducted with the young Finns population showed IDO enzyme’s involvement in the immune regulation of early atherosclerosis (35). A significant positive correlation of IDO activity in serum was observed among the patients with more advanced atherosclerosis, which suggest that activated KP may play a crucial role in vascular diseases (36). We previously showed that angiotensin II (AngII) infusion activates IFNγ in immune cells, which induces the expression of IDO1 and Kynu and increases 3-HAA production in the plasma and aortas of (Apolipoprotein E) Apoe^-/-^ mice, but not in Apoe^-/-^ IDO^-/-^ mice (37). Silencing of Kynu reduces the production of 3-HAA and further limits the production of matrix metallopeptidase-2 (MMP2) in SMCs, resulting in reduced AAA formation in Apoe^-/-^ mice (37). We also showed that AngII triggers the conversion of Trp to the following product, 3-HK and activates the generation of NAD(P)H oxidase (NOX)–mediated superoxide anions in endothelial cells. The superoxide could accelerate the apoptotic process in endothelial cells, leading to endothelial dysfunction (38). Kyn also intensifies certain MMPs *via* the MEK-Erk1/2 signaling pathway (39), which is important in vascular disease development (40). Measurement of Trp degradation and the product/substrate ratio (Kyn or 3-HK or 3-HAA/Trp) will contribute to a better understanding of the interplay between inflammation and vascular diseases (22). As above discussed, the Trp metabolism is modulated by many risk factors during vascular disease developments, which will be discussed with more evidence in the following sections.

### Inflammation Links KP Metabolism and Aortic Diseases

Cytokines are crucial mediators of inflammation and essential regulators of various immune and nonimmune cells in the aortic wall. The expression of IDO2 is basal, whereas that of IDO1, Kynu, etc, are induced by cytokines. A well-known cytokine is IFNγ, the most potent modulator of KP *in vitro* and *in vivo* models and humans (41). Activated IDO along with activated inflammatory parameters like IFNγ have a positive correlation with systemic chronic low-grade inflammation. However, LPS is not a strong inducer, it is also known to induce IDO. These findings reveal a direct link between the regulation of the KP and inflammation under aortic disease conditions. However, the functional contributions of secreted kynurenines by other cell types including neutrophils, monocyte/macrophages, mast cells, adipocytes, and platelets, remained to be determined. The following sections will provide some evidence on the role of these cells in aortic diseases and prove how kynurenine metabolism affects the inflammatory process (**Table 1**).

**Table 1:** Kynurenine metabolic members associated with aortic phenotype.

Aortic risk factors	Altering catabolites	Function	Associated disease	References
MMPs	3HAA ↑	ECM degradation	Aneurysm	(37)
SMC apoptosis	3HAA ↑	Cell inflammation	Aneurysm	(37)
EC apoptosis	3-HK ↑	EC dysfunction	Endothelial dysfunction	(38)
ROS, NADPH activation	3-HK ↑	Inhibits endothelial function	Endothelial dysfunction	(38)
Inhibition of BH_4_ synthesis	XA ↑	Inhibits BH_4_	May impair NO synthesis	(42, 43)
Macrophage apoptosis	3HAA	lowers plasma lipids and decreases atherosclerosis	Decrease atherosclerosis	(44)
EC function	Kyn ↑	Endothelium-derived relaxing factor	Sepsis	(45)
Atherosclerosis	Kyn↑	Suppression of T cells and possible protection against atherosclerosis.	Atherosclerosis	(46)
Atherosclerosis	3HAA	3HAA supplementation or HAAO inhibition both reduced atherosclerosis	Atherosclerosis	(47)

↑ denotes “increased Level of particular catabolite”.

### Kynurenines Activate Inflammatory Genes

Kyn was shown to be a proinflammatory metabolite. The increased Kyn was accompanied by the Nod-like receptor protein 2 (NLPR2) inflammasome expression and activation (48). This was also evidenced by increased caspase-1 expression and IL-1β release. After Kyn treatment, nuclear factor kappa-B (NF-κB) could translocate into the nucleus and binds to the promoter of NLRP2, subsequently increased NLRP2 transcription *in vitro* (48). To examine the IDO1 associated transcription, a comparative transcriptome analysis was performed between *Ido1^-/-^* and *Ido1^+/+^* rodent colon samples. Transcriptome analyses revealed that absence of IDO1 significantly down-regulated the pathways involving TLR and NF-kB signalings. Furthermore, dramatic changes in TLR and NF-kB signaling resulted in substantial changes in the expression of many inflammatory cytokines and chemokines (49). Similarly, 3-HK, and 3HAA both were reported to activate NF-kB signaling and mediate the EC apoptosis and SMC senescence respectively (37, 38, 50).

Many of the other kynurenines were also reported to modulate AhR, both at transcriptional as well at activity levels. AhR activation can influence inflammation and gene transcription through cross-regulation of many inflammatory signaling pathways. AhR activation was associated with activation of Toll-like receptor 2 (TLR2) and its downstream of NF-κB and the MAPKs, signaling pathways. Further, AhR activation also promotes phosphorylation of p65/NF-κB, JNK/MAPK, p38/MAPK, and ERK/MAPK pathways, which could further promote production of pro-inflammatory mediators including interleukin- 1β (IL-1β) and interleukin- 6 (IL-6) (51). Taken together these results demonstrate that the kynurenines including Kyn, 3-HK, and 3HAA (37, 38) are the molecular regulators of inflammation that can influence vascular inflammation.

## KP Regulation in Different Cell Types

### KP Regulation in Macrophages, Dendritic Cells and Neutrophils

Numerous studies demonstrated the crucial roles of inflammatory cells, including macrophages, dendritic cells and neutrophils for their contribution to the development of AAA (52). Aneurysm formation is associated with an accumulation of macrophages within the adventitia and the media. Monocytes/macrophages secrete TNFα, IFNγ and IL-6 inflammatory cytokines in the media and adventitia of aneurysmatic vessels (53). Under certain conditions, activated inflammatory macrophages express IDO and actively deplete their own Trp supply. In human macrophages and microglia cells, IFN-γ enhances the expression and activity of KMO (50, 54). A robust increase in KMO expression is associated with high levels of TNF-α and IL-6 following a systemic inflammatory challenge (55).

Moreover, accumulating evidence indicates that DCs can also induce tolerance, rather than immune activation, to the antigens they present. Similar to promoting immunity, promoting tolerance requires integrating information that DCs gather from the innate and adaptive immune systems. Plasmacytoid dendritic cells (pDCs) can produce type I interferons, such as IFN*α* and IFN*β*, to promote proinflammatory responses by activating effector T cell, cytotoxic T cells, and NK cells and can further facilitate AAA development (52, 56, 57). Dendritic cells (DCs) respond actively to tolerogenic signals, such as transforming growth factor-*β* (TGF-*β*), which regulates Trp metabolism. Staining of the aneurysm aortas with a marker of activated DCs, CD83, showed rare CD83 cells located at the adventitial/medial border, whereas those cells were not found in control aortas (58). DCs have been shown to mediate immunoregulation contributed by Trp catabolism. A study conducted by Braidy et al. demonstrated the KP activation in human monocyte-derived DCs (MDDCs) compared to the human primary macrophages using mRNA expression assays, high-performance liquid chromatography, mass spectrometry, and immunocytochemistry. Following activation of the KP using IFN*γ*, MDDCs can mediate apoptosis of Th cells *in vitro* (59). KAT, kynurenine 3‐hydroxylase, and 3‐hydroxyanthranilic acid dioxygenase (HAAO) appeared to be constitutively expressed in murine macrophages. Whereas the kynurenine 3‐hydroxylase and Kynu activity alone need IFN‐γ stimulation for their expression (60). IDO1 has been suggested to play a protective role in atherosclerosis due to its potential immunomodulatory effect (61). IDO1 is expressed in human atherosclerosis where it co-localizes with macrophages (46). In the murine systems, the absence of IDO1 shown to protect against atherosclerosis. Thus, Metghalchi *et al.* addressed the direct role of IDO1 in the modulation of immuno-inflammatory responses and its potential impact on the development of atherosclerosis. They also showed IDO1 expression co-localized with macrophages and SMCs in the aortic sinus of low-density lipoprotein receptor knock out (*Ldlr^−/−^)* mice (62). On the other hand, high fat diet (HFD) dramatically increases IDO activity in macrophages and VSMC of aortic sinus and circulating levels of KA and QA in atherosclerosis-prone *Ldlr^−/−^* mice compared with the chow diet. A marginal increase of transcriptional expression of IDO1 and increased protein levels were observed in peritoneal macrophages after the LPS challenge (63). These results indicate that Kyn and 3-HAA produced by macrophages are independently associated with vascular inflammation, suggesting a connection between macrophage produced Kynu and arterial remodeling.

Peripheral blood of aortic dissection patients showed a significant reduction in total lymphocytes, T lymphocytes, and T helper fractions, with a substantial increase in neutrophils (64) that shows neutrophils must have a critical role in aortic pathogenesis. During the AAA progression period, initially neutrophils stimulate a network of immune cell types that together can direct a chronic pathological response (65). Activated neutrophils form neutrophil extracellular traps (NETs), propagating the inflammatory reactions and culminating in eventual AAA (56). Neutrophils are also known to secrete ECM-degrading collagenases such as MMP-8 and certain proteases (66). In angiotensin II-lysyl oxidase inhibitor (*β-*Aminopropionitrile monofumarate; BAPN)–preconditioned aortic dissection model mice, adventitial neutrophil recruitment and activation were detected. Furthermore, it was confirmed that neutrophil-derived IL-6 enhances the adventitial inflammation, leading to aortic rupture (67). Besides, kynurenines such as 3HAA and 3HK are toxic and can trigger apoptosis in certain cell types (37, 38). Thus, it could be possible that immune infiltrates are present in the aortas of patients with medial degeneration could contribute to the local expression of death-promoting mediators in the diseased aortas. Accumulating data also shows that these MMPs secretion in the AAA wall (68) are controlled by inflammatory kynurenines (37, 38). MMPs were thought to be secreted essentially by only mesenchymal and monocyte/macrophage lineages. However, the neutrophil has now been recognized as a significant cell type that secretes these enzymes (67). Neutrophils are thus the vital source of MMP-2 and MMP-9, two matrix-degrading enzymes known to be critical in the formation of AAA by regulating KP metabolism.

### KP in Vascular Cells and Its Impact in Aortic Diseases

In many ways, endothelial and smooth muscle cell functions are linked to the health of the aorta (69). The widespread mechanisms link endothelial functions and aortic phenotypes are metalloproteinases and collagenase activation, collagen production and lysis, median and adventitial degradation, elastin lysis, and hypertension (70). In addition, endothelial cells respond to several stimulating factors, including smoking, hypertension and AT1 receptor stimulation and non-uniform distribution of the aortic wall (70). Besides, vascular smooth cells transformation and apoptosis also play a critical role in determining aortic health. The elaboration of cytokines, such as IL-2, IFNγ, and TNFα by a predominantly Th1 mononuclear response, stimulates proinflammatory osteopontin secretion from macrophages and vascular smooth muscle cells that further propagate the inflammatory response (15). Thus, the vascular phenotype majorly determined by the function and transformation of vascular cells. Despite the very first product of the kynurenine pathway, Kyn is a potential contributor to vessel relaxation; the other products are being studied for their involvement in vascular pathogenesis. Notable reports from our lab demonstrated the participation of kynurenine metabolism on endothelial dysfunction and aneurysm (37, 38) (**Table 1**).

Endothelial dysfunction and endothelial apoptosis are important factors in many aortic diseases’ pathogenesis including aortic aneurysm. Our group demonstrated the vasoactive peptide Ang II to induce vascular contractility, EC apoptosis, and dysfunction by mediating the activation of oxidative stress. We found that Trp catabolite, 3-HK mediates Ang II-induced EC apoptosis and subsequently endothelial dysfunction *via* the activation of NOX-derived superoxide anions *in vivo*. We further demonstrated that *Ido1* silencing could block the effect of Ang II action on endothelium, resulting in normal endothelial function (38). Wang et al., showed that the metabolism of tryptophan to kynurenine by IDO expressed in endothelial cells contributes to arterial vessel relaxation and blood pressure control (45). Sepiapterin reductase (SPR), which is one of the crucial enzymes involves in the *de novo* synthesis of tetrahydrobiopterin (BH_4_) (42). This BH_4_ acts as a critical regulator of endothelial nitric oxide synthase (eNOS) function and suggests that BH_4_ is a rational therapeutic target in vascular disease states, particularly for hypertension (71). Several findings confirmed a causal link between eNOS uncoupling and BH_4_ deficiency in AAA formation (72–74). SPR activity was reported to be inhibited by XA one of the KP metabolites (43), which indicates that elevated XA arising out of upregulated KP could attenuate BH_4_ biosynthesis and consequently EC dysfunction. On the other hand, reduced bioavailability of the BH_4_ also leads to dysregulated eNOS that could increase superoxide $\left({\text{O}}_{2}^{-}\right)$ production, which reacts with nitric oxide (NO) to generate peroxynitrite (75). Peroxynitrite consequently can nitrate IDO at Tyr15, Tyr345, and Tyr353, and inactivates IDO (76), which further leads to reduced production of kynurenine.

**Figure 2:**
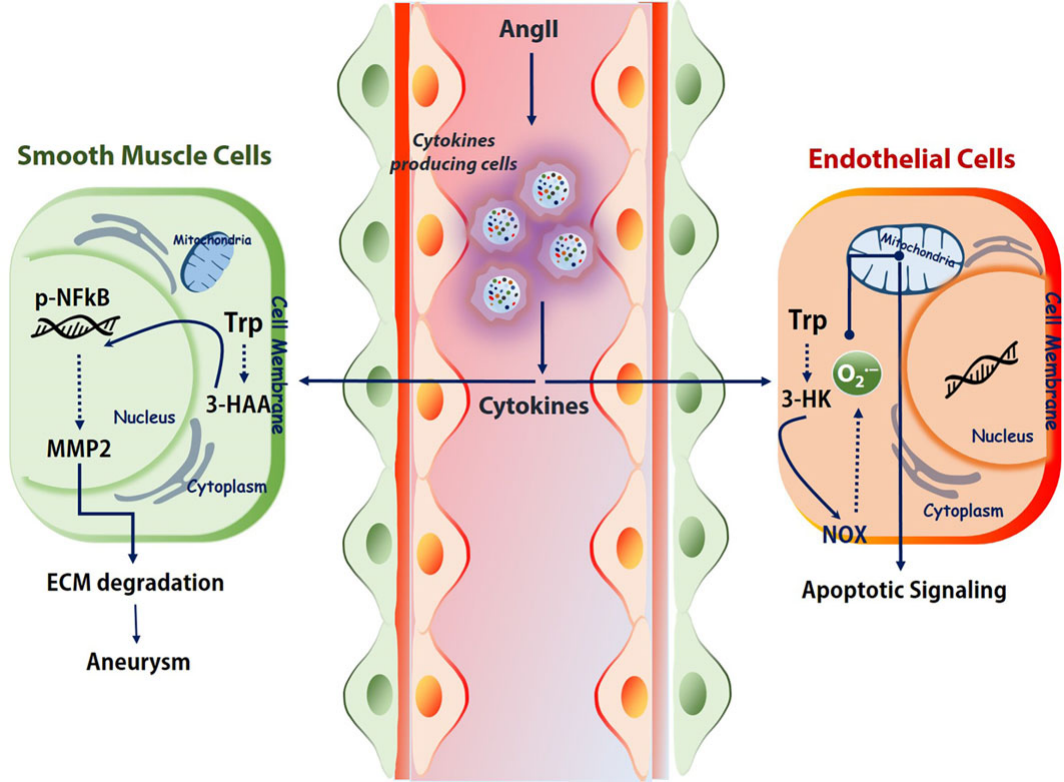
Kynurenines association to vascular diseases. Immune cells under challenged conditions release various cytokines (IFNγ, TGF-β, etc.) to regulate the activation and expression of the kynurenine pathway. Depends on the cell types and milieu, the activated KP effects differently. For example, in SMCs, 3-HAA triggers NF-κB and MMPs, which further degrades ECM. In endothelial cells, tryptophan catabolite 3-HK activates the NOX and produces superoxide. This superoxide further shoots up the apoptotic signaling. Trp, tryptophan; 3-HAA, 3-hydroxyanthranilic acid; AngII, angiotensin II; ECM, extracellular matrix; NOX, NADPH oxidase. 3-HK-3-hydroxykynurenine.

Some important cytokines like, IFNγ, usually show elevated level either in Ang II-treated mice or AAA patients (74). Studies from our group demonstrated a detrimental role of Kynu produced 3HAA in the pathogenesis of AAA in an AngII-Apoe^-/-^ animal model. Intra-peritoneal injections of 3-HAA for 6 weeks increased the expression and activity of MMP2 in aortas without affecting metabolic parameters. The acute infusion of AngII markedly increased the incidence of AAA in Apoe^−/−^ mice, but not in Apoe^−/−^IDO^−/−^ mice, which presented decreased elastic lamina degradation and aortic expansion. Findings from another group also showed an enhanced survival of VSMC when *Ido1* is silenced in murine model systems fed with HFD and either after infusion of AngII (dissecting AAA) or after topical peri-aortic elastase (non-dissecting AAA) (77). Mechanistically, 3HAA exposure in SMC mediates the NF-κB activation and further instigates the MMP2 upregulation (**Figure 2**). Hence, IDO1 deficiency can mitigate MMP2 upregulation in AAA model mice.

AAA is also an age-associated disease and the Trp pathway alters during aging (78, 79). Upregulation of KP in aging is due to IDO activation by age-related chronic inflammation (22). One key upstream mechanism that appears to target several pathways with age is kynurenine, a tryptophan metabolite and an endogenous aryl hydrocarbon receptor (AhR) agonist. The AhR signaling pathway has been reported to promote aging phenotypes across species and in different tissues (78, 80). Thus, mitigation of target receptors could prevent the kynurenine-induced increase in senescence-associated β-galactosidase and p21 levels and block aggregation of nuclear H3K9me3 (Histone 3 lysine 9 trimethylation) (79). Cellular senescence has historically been viewed as an irreversible cell-cycle arrest mechanism with complex biological processes such as development, tissue repair, ageing, and age-related disorders like aortic aneurysms. Thus, it is well understood that kynurenine metabolism involves triggering the senescence of vascular cells and targeting and controlling the activation of tryptophan metabolism may limit the development of AAA. Overall, casual relationships were well established between Kyn pathway and the development of AAA. Clinically, determining the Kyn or 3-HAA level at early stages in human patients could suggest that tryptophan-derived metabolites could be used as an early biomarker to identify AAA and atherosclerosis.

As reported above, many of these inflammatory signaling proteins are essential for cell cycle regulation. Hence, considerable enthusiasm remains for further investigations in this area, as well as it is yet to study using the KP pharmacological modulators for these KP enzyme proteins. Hence, it might be worth exploring the possible impact of modulating KP, which are regulated by cytokines for treating aortic diseases.

## Targeting KP as a Therapeutic Target for Aortic Diseases

Despite, there is no clinical trial was carried out with KP inhibitors to target the vascular diseases, KP activation has been observed in inflammation-related vascular diseases, such as atherosclerosis, AAA, and endothelial dysfunction. Many of the available KP inhibitors are known to inhibit inflammation during *in vivo* experiments. For example, *in vivo* experiments using animals have demonstrated that targeting IDO1, KMO, KYNU, and KAT II KP enzymes can regress cardiovascular diseases by reducing inflammation (22). Hence, pharmacological manipulation of the KP enzymes employing the drugs based on structures becomes an attractive drug development area. Thus, we may expect the emergence of kynurenines enzyme based modulators in future. In the following sections, we briefly outline some KP modulators tested at pre-clinical and clinical levels.

### IDO1 and TDO Inhibition and Its Effect on Reducing Inflammation

**Figure 3:**
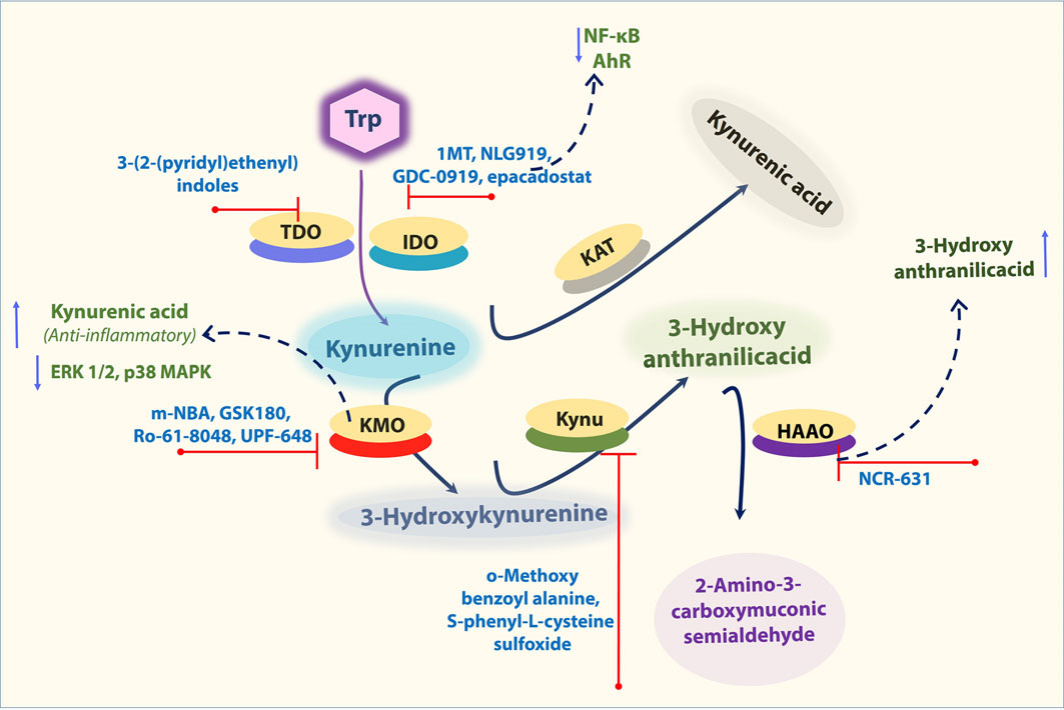
Important KP enzymes and their Inhibitors. Figure explains some KP modulators tested at pre-clinical and clinical levels. Refer to the text for the expanded form of abbreviations.

A well-known IDO1 inhibitor used clinically is 1-MT (referred to as Indoximod), the first and widely used competitive inhibitor of IDO1. Other notable IDO1 inhibitors are INCB024360 and NLG919 (an imidazoleisoindole derivative). NLG919 a potent direct small molecule IDO1 inhibitor, was tested in clinical trials (81). In another study, navoximod (GDC-0919, NLG-919) intervention in patients with tumor showed transiently decreased plasma kynurenine from baseline levels with kinetics consistent with its half-life (82, 83). TDO is also actively being tested to use as a target for cancer (84). The indole structure (3-(2-(pyridyl)ethenyl)indoles) based TDO inhibitor had proven pharmacokinetic profile and was tested for preclinical evaluation in cancer patients (85). However, it should be taken into consideration that systemic TDO inhibition will result in increased levels of TRP metabolites such as KYN due to increased availability of TRP for IDO1 as observed in the TDO-deficient mice (84). Depends on the environment and cell types Ido1 deficiency as well as IDO1 inhibition, is known to enhance the atherogenesis. However, the IDO1 inhibitor epacadostat has contrasting effects on macrophages, which could reduce the tissue factor (TF). IDO1 expressed in coronary atherosclerotic plaques was reported to contribute to thrombus formation by upregulated expression of TF in activated macrophages. The IDO1 inhibition by epacadostat, significantly reduced the TF expression by reducing the Kyn/Trp ratio and activity, as well as NF-κB (p65) binding activity in activated macrophages. Further, epacadostat could inhibit the aryl hydrocarbon receptor (or binding of Kyn to AhR) and reduced Kyn-induced TF expression in activated macrophages (25). In another way, the inhibition of AHR by its specific inhibitor CH223191 also significantly inhibited Kyn-induced TF expression in activated macrophages (25) (**Figure 3**). Besides, recent research also indicates that 1-MT induces an increase of KYNA in *ex vivo* and *in vivo*. Consistently, IDO^−/−^ mice also showed an increase of KYNA as Ido1 deficiency promotes a shift toward this branch of the kynurenine pathway (KP), which may be one potential mode of action by 1-MT and should be considered for further applications (86). These findings collectively suggest that the IDO1-mediated Kyn pathway plays a significant role in aortic diseases, and this area needs more study to decide pharmacological modulation of Ido1 and TDO enzyme activities to cure vascular inflammation associated diseases.

### Other KP Enzyme Inhibition for Reducing Vascular Inflammation

KMO expression was shown to be upregulated in response to challenging inflammatory conditions (55). Hence, KMO inhibition has been recognized as a potential therapeutic strategy to ameliorate inflammatory diseases in several animal models (87–89). From a therapeutic point of view, KMO is located at a critical branching point in the KP, its activity mediating opposite effects in the levels of 3-HK and QA *versus* KYNA.

KMO inhibitors based on the structure of the natural substrate KYN is m-Nitrobenzoylalanine (m-NBA), has an IC50 of 0.9 μM against KMO (90). Intraperitoneal administration (400 mg/kg) of this compound in rodents decreased the levels of 3-HK while increasing the levels of KYNA. Thus, inhibition of KMO should shunt the pathway away from the toxic metabolites 3-HK and QA and toward the formation of the protective metabolite KYNA (91). KYNA was assumed to have role as anti-inflammatory. KYNA decreased phosphorylation of extracellular signal-regulated kinases (ERK) 1/2, p38 MAPK, and Akt in colon epithelial cells. Further, it was also found to induce the accumulation of β-catenin. MAPK, PI3K/Akt and β-catenin pathways are well-known targets of GPR signaling (87, 88) (**Figure 3**). Therefore, it is possible that the observed inhibition of ERK and p38 and the induction of β-catenin accumulation after KYNA treatment are a consequence of GRP35 activation. Interestingly, all of these described effects of KYNA–GPR35 signaling might lead to the suppression or limitation of inflammation (92). Thus, inhibition of KMO can shunt the pathway away from the toxic metabolites 3-HK and QA and toward the formation of the protective metabolite KYNA. Similarly, many other KP enzyme inhibitors including, KMO inhibitors (GSK180, Ro-61-8048, UPF-648), KYNU inhibitors (o-Methoxybenzoyalanine, S-phenyl-L-cysteine sulfoxide), KAT inhibitors (PF-04859989, BFF 122) were also tested for their efficiency to reduce inflammatory diseases (22). In addition, Swainson et al. found that KMO inhibition using CHDI-340246 decreased acute simian immunodeficiency virus infection-induced increases in plasma levels of cytotoxic 3-HK and QA, and improved clinical outcomes as indicated by increased CD4+ T cell count and body weight (93). Despite this, the role of KMO inhibition in CVD is still unclear. Recently, Masanori Nishimura *et al.* found that both gene and protein expression levels of KMO were upregulated in macrophages in atherosclerotic aneurysmal samples obtained from patients (94). In another experiment, oxazolidinone GSK180 (3-(5,6-dichloro-2-oxobenzo[d]oxa-zol-3(2 H)-yl)propanoic acid; a KMO inhibitor), was shown to prevent extrapancreatic tissue injury to the many organs (88). However, further *in vivo* experiments using KMO inhibitor or knockout animals will help clarify the effects and potential mechanism of KMO in aortic aneurysms and related aortic diseases.

It was shown that 3-HAA, can modulate vascular inflammation and lipid metabolism. Supplementation of 3HAA reduced the athero formation (44) and blocking of HAAO (that catabolize 3HAA into other derivatives) by NCR-631 increased endogenous levels of 3-HAA, which reduced atherosclerosis (47) (**Figure 3**). Though many of these inhibitors were validated pre-clinically, their efficiency of inhibition of KP enzymes for vascular diseases and their application in the vascular field is largely unexplored. Understanding the outcome of multiple levels of KP inhibition would help us identify a potential target that would provide us with a choice to cure aortic diseases.

## Conclusions and Clinical Perspectives

As per our current knowledge, in the United States, out of >200 000 new patients diagnosed for AAA, >40 000 patients are undergoing highly morbid aortic reconstructions. This approach is a catastrophic event associated with near-certain mortality, and no pharmaceutical currently exists to slow aneurysm growth (95). Evidence shows that targeting inflammation in vascular diseases could reduce secondary cardiovascular events (96).

Evidence-based studies confirmed the perturbed tryptophan metabolism and its association with many aortic diseases, particularly atherosclerosis and AAA. AAA most likely associated to the perturbed cytokine levels, which is linked to the disturbed tryptophan metabolism. Cytokines lead generation of kynurenines in immune or vascular cells, further triggers vascular inflammation. This metabolic behavior shows many commonalities to share with other vascular immune disorders. Generally, chronic inflammation drives initial aortic ectasia and dilation, and later, the tension on the aortic wall continues to expand. When wall tension drives sac expansion, no medical intervention will work except surgical aortic reconstruction (95). Hence, we may assume that limiting the inflammation-mediated downstream mechanisms, particularly the kynurenine pathway, emphasizes a further trend and application of these interventions.

Furthermore, as the product to substrate ratio of tryptophan metabolism is an indicator of vascular inflammation, controlling the patients’ Trp metabolic profile could be viable if anyone is diagnosed earlier. This will also allow early identification of patients at risk of vascular diseases that could be crucial to the success of nonsurgical treatment.

## Author Contributions

TR: Conceived idea and wrote the manuscript. Y-MH, DZ, and C-JY: Revised the manuscript. M-HZ: Conceived idea and revised the manuscript. All authors contributed to the article and approved the submitted version.

## Funding

This work was supported by the AHA postdoctoral fellowship award: 19POST34380156 to TR. M-HZ is a recipient of the National Established Investigator Award of the American Heart Association.

## Conflict of Interest

The authors declare that the research was conducted in the absence of any commercial or financial relationships that could be construed as a potential conflict of interest.

## Publisher’s Note

All claims expressed in this article are solely those of the authors and do not necessarily represent those of their affiliated organizations, or those of the publisher, the editors and the reviewers. Any product that may be evaluated in this article, or claim that may be made by its manufacturer, is not guaranteed or endorsed by the publisher.

## Abbreviations

3-HAA, 3-hydroxyanthranilic acid; 3-HK, 3-hydroxykynurenine; AA, anthranilic acid; AAA, Abdominal aortic aneurysm; AhR, aryl hydrocarbon receptor; AngII, Angiotensin II; Apoe, Apolipoprotein E; BAPN, *β*-Aminopropionitrile monofumarate; BH_4_, tetrahydrobiopterin; CAD, coronary artery disease; EC, endothelial cells; ECM, extracellular matrix; eNOS, endothelial nitric oxide synthase; H3K9me3, Histone 3 lysine 9 trimethylation; HAAO, 3‐hydroxyanthranilic acid dioxygenase; HFD, high fat diet; IDO, Indoleamine 2, 3-dioxygenase; IFNγ, interferon-gamma; IL-1, interleukin 1; IL-6, interleukin 6; KA, kynurenic acid; KAT, kynurenine aminotransferase; KMO, Kynurenine-3-monooxygenase; KP, Kynurenine pathway; Kyn, kynurenine; Kynu, kynureninase; Ldlr, low-density lipoprotein receptor; LKO, L-selectin-knockout mice; LPS, lipopolysaccharide; MDDCs, monocyte-derived DCs; MMP, Matrix metalloproteinase; m-NBA, Nitrobenzoylalanine; NAD^+^, Nicotinamide adenine dinucleotide^+^; NETs, neutrophil extracellular traps; NF-κB, nuclear factor kappa-B; NLPR2, Nod-like receptor protein 2; NO, nitric oxide; NOX, NADPH oxidase; ${\text{O}}_{2}^{-}$ , superoxide; O-MBA, ortho methoxy benzoyl alanine; pDC, Plasmacytoid dendritic cells; PMN, polymorphonuclear neutrophils; PVAT, perivascular aortic tissue; QA, quinolinic acid; QPRT, Quinolinate phosphoribosyl transferase; SPR, Sepiapterin reductase; TAA, Thoracic aortic aneurysm; TDO, tryptophan-2,3-dioxygenase; TGF-β, transforming growth factor-β; TLR2, Toll-like receptor 2; TNFα, tumor necrosis factor-alpha; Trp, Tryptophan; VSMC, vascular smooth muscle cells; XA, xanthurenic acid
